# Variation in Genomic Methylation in Natural Populations of Chinese White Poplar

**DOI:** 10.1371/journal.pone.0063977

**Published:** 2013-05-21

**Authors:** Kaifeng Ma, Yuepeng Song, Xiaohui Yang, Zhiyi Zhang, Deqiang Zhang

**Affiliations:** 1 National Engineering Laboratory for Tree Breeding, College of Biological Sciences and Technology, Beijing Forestry University, Beijing, P.R. China; 2 Key Laboratory of Genetics and Breeding in Forest Trees and Ornamental Plants, Ministry of Education, College of Biological Sciences and Technology, Beijing Forestry University, Beijing, P.R. China; National Taiwan University, Taiwan

## Abstract

**Background:**

It is thought that methylcytosine can be inherited through meiosis and mitosis, and that epigenetic variation may be under genetic control or correlation may be caused by neutral drift. However, DNA methylation also varies with tissue, developmental stage, and environmental factors. Eliminating these factors, we analyzed the levels and patterns, diversity and structure of genomic methylcytosine in the xylem of nine natural populations of Chinese white poplar.

**Principal Findings:**

On average, the relative total methylation and non-methylation levels were approximately 26.567% and 42.708% (*P*<0.001), respectively. Also, the relative CNG methylation level was higher than the relative CG methylation level. The relative methylation/non-methylation levels were significantly different among the nine natural populations. Epigenetic diversity ranged from 0.811 (Gansu) to 1.211 (Shaanxi), and the coefficients of epigenetic differentiation (*G_ST_* = 0.159) were assessed by Shannon’s diversity index. Co-inertia analysis indicated that methylation-sensitive polymorphism (MSP) and genomic methylation pattern (CG-CNG) profiles gave similar distributions. Using a between-group eigen analysis, we found that the Hebei and Shanxi populations were independent of each other, but the Henan population intersected with the other populations, to some degree.

**Conclusions:**

Genome methylation in *Populus tomentosa* presented tissue-specific characteristics and the relative 5′-CCGG methylation level was higher in xylem than in leaves. Meanwhile, the genome methylation in the xylem shows great epigenetic variation and could be fixed and inherited though mitosis. Compared to genetic structure, data suggest that epigenetic and genetic variation do not completely match.

## Introduction

Epigenetic regulation, which is not based on differences in DNA sequence [1]–[3], plays important roles in genome protection, control of gene expression and nuclear inheritance via chromatin structural remodeling and is crucial for promoting phenotypic variation of organisms [4]. DNA methylation, which involves the addition of a methyl group (–CH_3_) from *S*-adenosyl-L-methionine to the 5-position of the cytosine pyrimidine ring or the number 6 nitrogen of the adenine purine ring [5], is one of the best-studied epigenetic mechanisms [6]. Cytosine methylation generally occurs in the symmetrical sequence CG, but can also be found in CNG or CNN sequences [7]–[9]. In plants, the genomic methylation level is about 6%–30% [10] and many methods have been exploited to detect genomic methylation [6], [11]. One powerful and highly stable tool is methylation sensitive amplification polymorphism (MSAP), established based on the amplified fragment length polymorphism (AFLP) [12], [13] technique, which was adapted for the analysis of genome-wide sequence-specific methylation status without a *priori* knowledge of the genome sequence [14], [15]. Nowadays, this technique is used widely to examine epigenetic variation in plants 12,16–18.

Genome sequence determines genetic diversity, which can be assessed at the molecular level by a variety of techniques, i.e., AFLP markers [19], [20] and SSR markers [21]. However, emerging evidence indicates that the DNA sequence variation is not the only determinant of phenotypic variation. For example, methylation polymorphism among varieties of cultivated rice [22] and variation among individuals in the degree of methylation of a gene, termed epialleles [23], produce novel phenotypes that are often stably transmitted through generations [24]–[27]. Therefore, genomic methylation can be used as a reliable molecular marker to identify the cultivated rice genotypes [24]. And further, researches on the methylation diversity and epigenetic variation begin to attract focus in *Arabidopsis thaliana* [12], cotton [16], and mangrove [17].

The Chinese white poplar (*Populus tomentosa* Carr., 2*n = *2*x = *38), a perennial tree that is cultivated for commercial timber production and plays an important role in ecological and environmental protection along the Yellow River in China [28], [29], belongs to the *Populus* Section Duby and has given rise to many ecotypes during the evolution of the species. Genetic diversity and population structure in *P*. *tomentosa* have been investigated [21]; however, little knowledge is known about the genomic methylation diversity and epigenetic structure of natural populations of *P*. *tomentosa*. Although previous research [18] has discussed association analysis between relative methylation levels, methylation patterns and phenotypes in a hybridization population, variation in genomic methylation in natural populations remains unclear. Here, we examined the epigenetic diversity and structure in nine natural populations of *P*. *tomentosa* by using MSAP markers and multivariate statistical analysis.

## Results

### Polymorphic MSAP Bands

**Figure 1:**
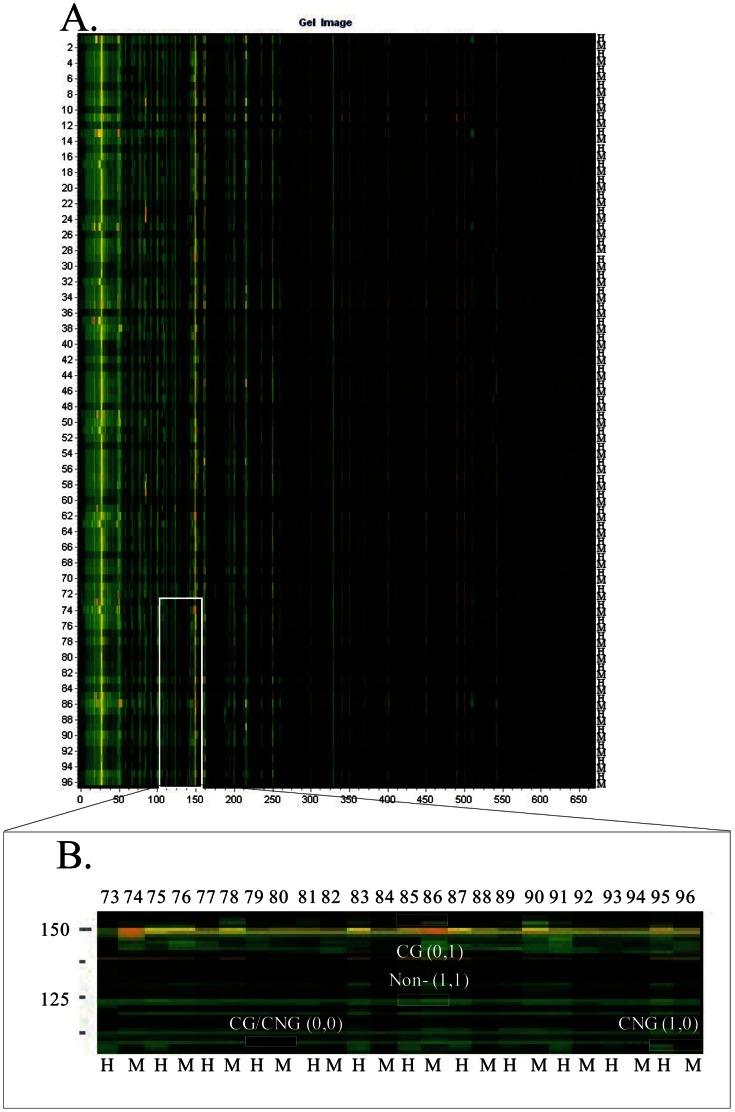
Methylation sensitive amplification polymorphism analysis of natural populations of *P. tomentosa*. Gel image generated by GeneMarker1.71 software after selective amplification products separated by capillary electrophoresis and visualized by fluorescent detection. The primer-pair combination E(ATT)+H/M(ATC) was used for amplification. Green bands showed amplification products and orange bands showed standard samples. H and M represented 48 genomic DNA samples digested by *Eco*RI/*Hpa*II, and *Eco*RI*/Msp*I, respectively. (**A)** Gel image generated by GeneMarker1.71 software. The numbers on the left indicated lane numbers and the numbers below represented fragment length (bp). (**B**) Enlargement of one part of (**A**), rotated 90° counter clockwise, displaying four patterns of band combinations. CNG (1,0), CG (0,1), CG/CNG (0,0) and Non- (1,1) indicated hemi-methylation, full methylation, uninformative site, and non-methylation, respectively.

We used MSAP analysis to detect the methylation patterns of 432 individuals of *P. tomentosa*, which were divided into nine populations according to their geographic origins. Each of the 432 genomic DNA samples was double-digested with *Eco*RI/*Hpa*II and *Eco*RI/*Msp*I, respectively; *Hpa*II and *Msp*I have the same recognition site (5′-CCGG) and digest non-methylation sequence, but *Hpa*II will not cut if the internal C is full methylated [18]. After ligation of linkers and pre-amplification, 30 primer pair combinations were used for selective amplification and the resulting bands were visualized by capillary electrophoresis (CE) and GeneMarker V1.7.1 (Figure 1A). Disregarding bands under 60 bp for their fuzzy appearance, we obtained 2408 bands; out of these bands, 2393 (99.38%) were polymorphic (Table 1), with an average number of polymorphic bands of approximately 80 per primer pair combination. Primer-pair combination E(AAT)-H/M(TCT) produced the fewest polymorphic bands (41) and E(TCT)-H/M(CTC) produced the most polymorphic bands (114).

**Table 1:** Polymorphic bands generated by 30 primer-pair combinations for selective amplification.

Primer-pair combinations	Number of polymorphic markers	Range of polymorphic fragment length (bp)
E(ATT)-H/M(ATC)	74	67–479
E(CAG)-H/M(ATC)	68	62–463
E(GAA)-H/M(CAA)	97	69–498
E(TAC)-H/M(CTC)	101	69–489
E(TCT)-H/M(CTC)	114	71–496
E(AAG)-H/M(TAT)	95	69–490
E(AAG)-H/M(AAA)	76	67–498
E(AAT)-H/M(AAA)	76	70–498
E(ACA)-H/M(AAG)	74	69–482
E(ACT)-H/M(AAG)	76	69–495
E(AGC)-H/M(AAT)	81	65–496
E(AGT)-H/M(AAT)	88	67–484
E(ATC)-H/M(ATT)	104	69–496
E(CCA)-H/M(ATT)	86	67–479
E(GAG)-H/M(CAA)	67	70–485
E(CCT)-H/M(GAA)	76	71–488
E(CTC)-H/M(GAA)	61	67–488
E(ACA)-H/M(GAG)	84	68–499
E(ACT)-H/M(GAG)	89	67–488
E(AGC)-H/M(TAC)	82	70–495
E(ATC)-H/M(TAC)	75	67–491
E(CAG)-H/M(TAT)	75	65–479
E(CCT)-H/M(TAT)	63	67–446
E(GAA)-H/M(TCT)	96	66–481
E(AAT)-H/M(TCT)	41	68–457
E(AGT)-H/M(TAC)	67	71–473
E(ATT)-H/M(GAA)	106	69–471
E(CCA)-H/M(CAA)	61	69–343
E(CTC)-H/M(ATC)	68	69–460
E(GAG)-H/M(AAG)	72	71–373
Total	2393	

### Relative Genomic Methylation Levels

MSAP detected four patterns of 5′-CCGG sites (Figure 1B), i.e., hemi-methylation (CNG methylation) (1,0), full methylation (CG methylation) (0,1), non-methylation (1,1), and uninformative site (0,0). We also defined the sum of hemi-methylation and full methylation as total methylation to explain the 5′-CCGG methylation sites. The relative methylation/non-methylation and uninformative sites levels were calculated as percentages of the different patterns’ marker amounts and the total markers, which were equal to the total number of all bands.

Within the 432 individuals of *P*. *tomentosa* (Table 2), the relative total methylation and non-methylation levels were 26.567±5.856% and 42.708±6.732%, respectively. The relative non-methylation level was significantly larger than the relative total methylation level as determined by a Wilcoxon rank sum test (*P*<0.001). Also, compared to the relative CG methylation level (13.101±2.281%), the relative CNG methylation level (13.466±4.644%) was larger (*P*<0.001).

**Table 2:** Relative genomic methylation/non-methylation levels in the natural populations of *Populus tomentosa.*

Pattern	Mean	SD	CV (%)	Max	Min	Significant difference among natural populations^a^		
						?^2^	*df*	*P*-value
CNG^b^	13.466	4.644	34.484	32.309	5.316	28.573	8	<0.001
CG^b^	13.101	2.281	17.410	26.578	8.721	30.542	8	<0.001
Total^c^	26.567	5.856	22.044	45.266	14.037	53.579	8	<0.001
Non-c	42.708	6.732	15.762	53.821	20.847	149.12	8	<0.001
Uninformative	30.725	4.542	14.784	61.088	23.588	79.770	8	<0.001

a The significance of differences among the nine natural populations was examined by a Kruskal–Wallis *H* test.

b Significant difference between relative CNG and CG methylation levels was examined using a Wilcoxon rank sum test (*P*<0.001).

c Significant difference between relative total methylation and non-methylation levels was examined using a Wilcoxon rank sum test (*P*<0.001).

**Figure 2:**
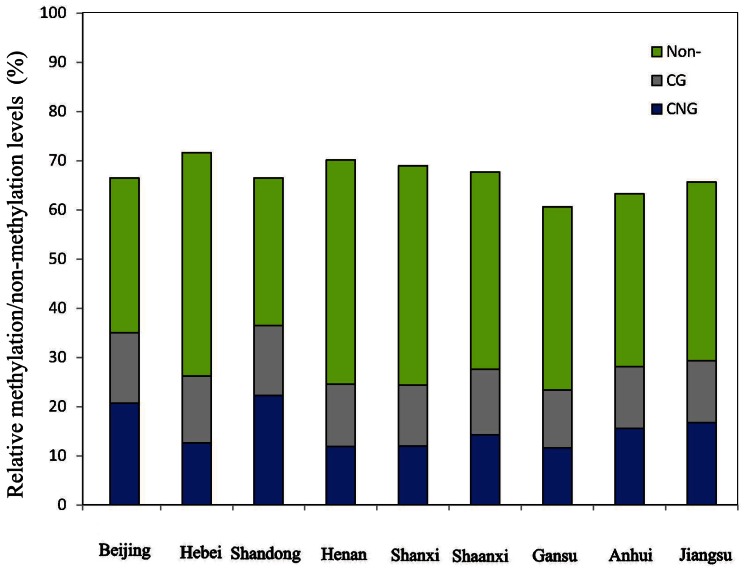
Relative methylation/non-methylation levels in the nine natural populations. CNG, CG, and Non- represented hemi-methylation, full methylation, and non-methylation, respectively. Significant differences between relative CNG and CG methylation levels within each population was examined using a Wilcoxon rank sum test with *P*-values of *P* = 0.026 (Beijing), *P* = 0.018 (Hebei), *P* = 0.002 (Shandong), *P* = 0.001(Henan), *P* = 0.225 (Shanxi), *P* = 0.086 (Shaanxi), *P* = 0.109 (Gansu), *P* = 0.821 (Anhui), and *P* = 0.602 (Jiangsu), respectively. Also, significant differences between relative total methylation and non-methylation levels within each population was also examined using the Wilcoxon rank sum test with *P*-values *P* = 0.066 (Beijing), *P* = 0.001 (Hebei), *P* = 0.012 (Shandong), *P*<0.001(Henan), *P*<0.001 (Shanxi), *P*<0.001 (Shaanxi), *P* = 0.055 (Gansu), *P* = 0.064 (Anhui), and *P* = 0.465 (Jiangsu), respectively.

Among the nine natural populations, the relative methylation/non-methylation levels were significantly different (*P*<0.001) examined by a Kruskal–Wallis *H* test (Figure 2, Table 2). The relative total methylation level (36.436±8.720%) and relative hemi-methylation level (22.268±7.423%) in the Shandong population were the greatest among the populations. The relative full methylation level in the population of Beijing (14.325±2.087%) displayed the greatest value and the largest relative non-methylation level (45.551±3.262%) was found in the population of Henan (Figure 2). We also found significant differences between relative CG methylation level and relative CNG methylation level within the populations of Beijing (*P* = 0.026), Hebei (*P* = 0.018), Shandong (*P* = 0.002) and Henan (*P* = 0.001), as well as significant differences between relative total methylation level and relative non-methylation level within the populations of Hebei (*P* = 0.001), Shandong (*P* = 0.012), Henan (*P*<0.001), Shanxi (*P*<0.001), Shaanxi (*P*<0.001), respectively (Figure 2).

### Diversity of Genome Methylation in the Natural Populations

We calculated Shannon’s diversity index, based on the frequency of the methylation patterns within each marker, to assess epigenetic diversity in the nine natural populations of *P*. *tomentosa*. The diversity of these populations was measured as 1.187±0.532 (Beijing), 1.149±0.558 (Hebei), 1.179±0.521 (Shandong), 1.087±0.584 (Henan), 1.081±0.586 (Shanxi), 1.211±0.556 (Shaanxi), 0.811±0.577 (Gansu), 1.063±0.536 (Anhui), and 0.915±0.561 (Jiangsu). These values were significantly different among the natural populations (Kruskal–Wallis χ^2^ = 1039.017, *P*<0.001). The Shaanxi population had the maximum value and thus it shows higher variation than other populations. However, the Gansu population displayed the least Shannon’s diversity. And the diversity, was equaled to 1.280, within the 432 individuals was computed at last. Because dominant MASP markers generated four patterns of methylation/non-methylation, we could not calculate deviation from the Hardy-Weinberg equilibrium. Thus, we computed the coefficients of epigenetic differentiation (*G_ST_*) relying on the Shannon’s diversity of the natural populations and found that the *G_ST_* values were distributed from 0.054 (Shaanxi) to 0.366 (Gansu) and a *G_ST_* = 0.159 was obtained overall (Table 3).

**Table 3:** The epigenetic diversity^a^ and coefficients of epigenetic differentiation (*G_ST_*)^b^ assessed by Shannon’s diversity index.

Population	Mean	SD	CV (%)	Max	Min	*G_ST_*
Beijing	1.187	0.532	44.820	1.985	0.000	0.073
Hebei	1.149	0.558	48.585	1.998	0.000	0.103
Shandong	1.179	0.521	44.208	1.994	0.000	0.079
Henan	1.087	0.584	53.693	1.998	0.000	0.150
Shanxi	1.081	0.586	54.227	1.997	0.000	0.155
Shaanxi	1.211	0.556	45.874	1.996	0.000	0.054
Gansu	0.811	0.577	71.162	1.918	0.000	0.366
Anhui	1.063	0.536	50.427	1.971	0.000	0.169
Jiangsu	0.915	0.561	61.263	1.922	0.000	0.285
Average	1.076	0.571	53.067	1.998	0.000	0.159
Total	1.280	0.503	39.289	2.000	0.024	

a The epigenetic diversity was assessed by Shannon’s diversity index: *I* = – ∑ *P_i_* log_2_ (*P_i_*). The difference of the index among natural populations was tested using a Kruskal–Wallis *H* test with the chi-square value = 1039.017 (*P*<0.001) that was adjusted by the sequential Bonferroni correction.

b *G_ST_* = (*H_total_* – *H_pop_*)/*H_total_*.

### Structures and Relationship of MSP and CG-CNG Profiles

**Figure 3:**
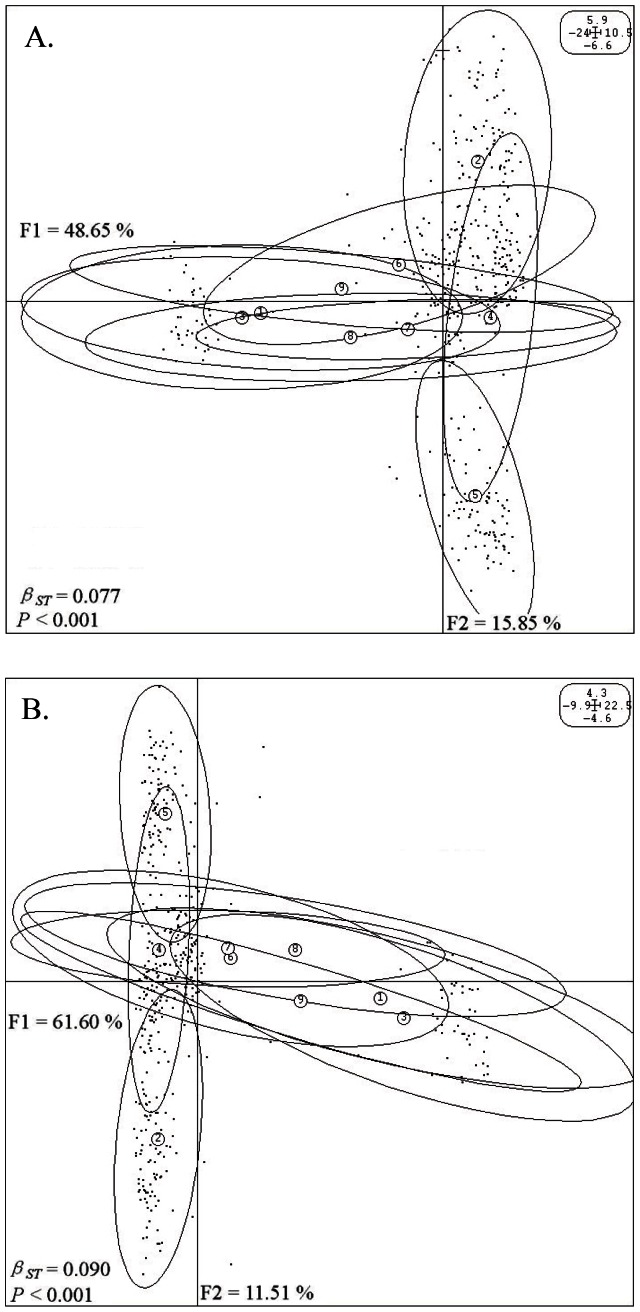
Eigen analysis between the nine natural populations of *P tomentosa* using PCA values based on epigenetic covariance matrices. F1 and F2 values showed the contribution of the first two principal components summarizing the total variance of each data set. Numbers within circles represented populations of: (1) Beijing, (2) Hebei, (3) Shandong, (4) Henan, (5) Shanxi, (6) Shaanxi, (7) Gansu, (8) Anhui, and (9) Jiangsu. Ellipses represented projection boundary of each population. *β_ST_* were calculated by BPCA for epigenetic profiles and tested with 9999 Romesburg randomization permutations. (**A**) Eigen analysis between the nine natural populations of *P*. *tomentosa* using PCA values based on the MSP matrix. (**B**) Eigen analysis between the nine natural populations of *P*. *tomentosa* using PCA values based on the CG-CNG matrix.

The axes chosen in order of PCA based on covariance matrices of MSP, and CG-CNG together should represent over 90% of the information contained in the two profiles (MSP matrix and CG-CNG matrix), respectively, were used to carry out between-group eigen analysis (BPCA-PCA among groups based on PCA among individuals). For the between-populations analysis of the MSP matrix, a significant *β_ST_*_ = _0.077 (*P*<0.001) (Figure 3A) showed that epigenetic variance could be partitioned into between- (7.700%) and within- (92.300%) populations components. Also, summarizing 64.50% of the total inertia, the nine populations were projected into one subspace. The Shanxi population was non-overlapping with other populations except for a partial intersection with the Henan population which also intersected with Hebei, Shaanxi, and other populations.

For the between-populations analysis of the CG-CNG matrix, a *β_ST_*_ = _0.090 (*P*<0.001) (Figure 3B) was computed, indicating that hemi-methylation and full methylation variance could also be partitioned into between- (9.000%) and within- (91.000%) populations components. The natural populations projected into a subspace with the first two axes explaining 73.11% of the variation in total inertia. The Henan population intersected with other populations to some degree, but the populations of Hebei and Shanxi were independent of each other.

**Figure 4:**
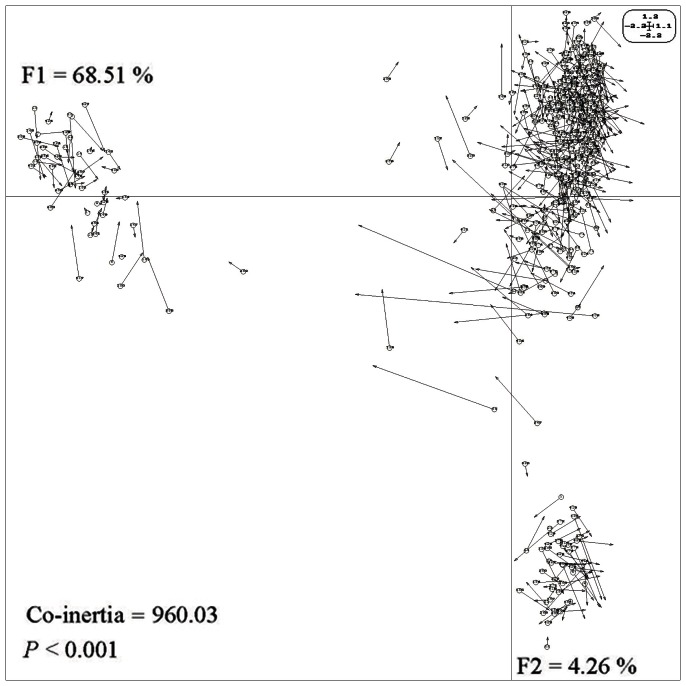
Co-inertia analysis of *P tomentosa* using PCA scores on the basis of MSP and CG-CNG covariance matrices, which would be maximized. F1 and F2 values showed the contribution of the two principal components summarizing the total variance of each data set. The circles corresponded to the projection of the CG-CNG data profiles and arrows indicated the MSP data profiles. Numbers from 1 to 432 represent populations of: Beijing (1–20), Hebei (21–134), Shandong (135–153), Henan (154–267), Shanxi (268–347), Shaanxi (348–411), Gansu (412–417), Anhui (418–427), and Jiangsu (428–432). The significance was tested with 9999 Romesburg randomization permutations.

The relationship, detected by using pairwise *β_ST_* (r = 0.945, *P*<0.001), between MSP and CG-CNG profiles was significantly correlated. We also evaluated the two profiles contributing to the structure of *P*. *tomentosa* populations using co-inertia analysis. We found that the two profiles gave similar distributions (Figure 4) and the first two axes explained 68.51%, and 4.26% of the total co-inertia (*P*<0.001), respectively, with a greater contribution from the CG-CNG profiles.

## Discussion

*P*. *tomentosa* is one of the main commercial tree species used for timber production, and its xylem was employed to explore genomic methylation in our study. We extracted genomic DNA from the xylem, not from fresh leaves or buds previously [18], [21] for two reasons. First, DNA extraction from timber yielding appropriate DNA quality for PCR amplification allows molecular genetic investigations of wood tissue [30], [31], which is the essential agricultural product of this species. Therefore, this analysis sets the stage for future examination of epigenetic regulation of wood traits. Second, genomic methylation levels and patterns can be tissue-specific in plants [15], [32], [33] and examination of a single tissue eliminated variation from tissue specificity.

Different methylation levels and patterns can be detected in different plant genomes. Also, the CG methylation level is high in all species, but the CNG methylation level is different among species [34]. In maize, the relative CG methylation was 16.33–16.89% and the CNG methylation was 8.63–9.79% at the 5′-CCGG sites [35]. Ma et al. found that CG methylation level (9.26±0.96%) was larger than CNG methylation level (8.61±1.10%) [18]. However, the data in this experiment showed that relative CNG methylation level was larger than relative CG methylation level and the relative non-methylation level was larger than the relative total methylation level in general.

Genomic methylation shows tissue and developmental stage specific characteristics [33], [36]–38. For example, Hauben et al. found that levels of methylcytosine from genomic DNA prepared from cotyledons and the fourth leaf were not the same [33]. Also, methylation levels and patterns in mature leaves, pericarp and locular tissue of tomato [37] displayed dynamic changes during fruit ripening. Moreover, the 5-methylcytosine percentages in adult chestnuts showed dynamic changes in degree during the active growth period and dormancy phase [38]. We used the newly formed xylem at 1.3 m of the trunk to prepare genome DNA to eliminate these specific characteristics and explored the relative total methylation level (26.567±5.856%), which was larger than the relative total methylation level in leaves according to previously published data (17.87±1.47%) [18].

Methylation status of 5′-CCGG sites was stable in *Arabidopsis thaliana* ecotypes, but it differed for 24–34% of the amplified fragments between different ecotypes [12], and methylation level in mangrove plants from riverside (32.1%) was greater than that from salt marsh (14.6%) [17]. Thus, it seems that environment can shape cytosine methylation. Similarly, methylation levels and patterns were significantly different among the nine natural populations of *P*. *tomentosa*. Moreover, Shannon’s diversity index, based on the frequency of different patterns in each polymorphic band among individuals, was used to assess epigenetic diversity in the natural populations and it showed that the diversity was significantly different among populations and ranged from 0.811 (Gansu) to 1.211 (Shaanxi). We also computed the total coefficient of epigenetic differentiation (*G_ST = _*0.159). Interestingly, our research uncovered substantial epigenetic diversity in natural populations, even though the experimental genotypes we used were propagated from roots [39] and planted in the same conditions, and moreover, DNA methylation status can be reversed [40]. In other words, can methylcytosine be inherited in future generations despite environmental factors in Chinese white poplar?

According to Wigler et al. [41], arbitrary patterns of methylcytosine in plasmids were stably maintained for many cell cycles after the plasmids were integrated into the genomes of transfected cells. Also, methylation patterns were maintained essentially unchanged for 80 cell divisions in a system that controlled for the effects of copy number and integration site [42], and in plants, DNA methylation is often heritable [24]–[27]. We detected that methylation-sensitive polymorphism (MSP) and methylation pattern (CG-CNG) profiles were significantly correlated and gave similar distributions, although CG-CNG profiles gave a greater contribution. Meanwhile, performing MSP and CG-CNG profiles structures with the between-group eigen analysis, we found significant differences among different populations eliminating environmental factors. Therefore, our results suggested that variation in genomic methylation can be fixed and inherited though mitosis in *P*. *tomentosa*.

In addition, Messeguer et al. [32] proposed that methylcytosine could be inherited through meiosis in a Mendelian fashion, and it is also suggested that epigenetic variation is under genetic control and/or their correlation was caused by neutral drift [43]. Genetic variation revealed by SSR markers was used to divide a population of 460 *P*. *tomentosa*, 432 of which were the same as individuals we used to perform the MSAP process. The SSR analysis divided the individuals into three subsets, providing reasonable support for the identified populations, i.e., the northeastern subset included Beijing, Shandong and Hebei, the southern subset included Henan, Shaanxi, Anhui, and Jiangsu, and the northwestern subset included Gansu, Ningxia and Shanxi. The southern region is probably the center of the current species distribution [21]. However, based on MSAP marker profiles, we found the populations of Hebei and Shanxi were independent of each other, and the Henan population, which displayed the maximum non-methylation level, intersected with other populations to some degree. We suggest that Henan, also the geographic center of the nine provinces, may be the center of the species distribution. The genetic and epigenetic population structures in Chinese white poplar were not in the same, indicating that there is greater epigenetic variation than genetic variation [17], for methylation variation induced by environment, the process of which can be a source of random variation in natural populations [44], can be maintained via mitosis.

## Materials and Methods

### Plant Materials

The nine natural populations of *P. tomentosa* were represented by a total of 432 individuals, each of which had three clones generated from root segments. Samples were collected from the *P. tomentosa* natural distribution range (nine municipalities and provinces of China, i.e., Beijing, Hebei, Shandong, Henan, Shanxi, Shaanxi, Gansu, Anhui, Jiangsu) covering an area of 1 million km^2^, in 1982. These plants were grown (4 m×4 m) in Guanxian County, Shandong Province (Figure S1). In 2011, using a sharp blade, we uncovered the bark (approximately 5 cm×5 cm) of the tree trunk at breast height and dug out part of the xylem (Figure S1). The material was divided into nine groups according to their region of origin and frozen quickly in liquid nitrogen for DNA extraction. This study was carried out in strict accordance with the recommendations in the Guide for Observational and Field Studies. All necessary permits were obtained for the described field studies. The sampling of all individuals of *P. tomentosa* was approved by Youhui Zhang, director of National Garden of *P. tomentosa* in Guan Xian County, Shandong Province.

### DNA Extraction

Plant materials were ground with liquid nitrogen and DNA was isolated using the CTAB method [45], detected by NanoVue UV/visible spectrophotometer (GE Healthcare Company) and stored at –20°C.

### Detection of Genomic Methylation

The processes, e.g. double digestion with restriction endonuclease combinations of *Eco*RI/*Hpa*II and *Eco*RI/*Msp*I, ligation, pre- and selective-amplification, etc., to detect the genomic methylation in the natural population were the same as described by Ma et al. [18]. However, the detection of methylation sites involved some differences: first, not all of the 30 primer-pair combinations for selective amplification were the same (Table 1); second, the products of selective amplification were resolved by capillary electrophoresis (CE) with fluorescent detection methods (Tsingke Company, Beijing, China) and bands were generated by GeneMarker V1.7.1. Also, these bands were transformed into a binary character matrix, using “0″ to define the absence and “1″ to define the presence.

### Statistical Analysis

Each genomic DNA sample was digested by *Eco*RI/*Hpa*II, and *Eco*RI/*Msp*I, separately and the methylation sensitive amplified polymorphism bands were transformed as “1″ or “0″ for the presence or absence, respectively. Then, four patterns, each of which corresponds to one condition of methylation/non-methylation, were displayed (Figure 1B): (1) present in *Eco*RI/*Hpa*II but absence in *Eco*RI/*Msp*I (1,0), hemi-methylation; (2) absent in *Eco*RI/*Hpa*II but present in *Eco*RI/*Msp*I (0,1), full methylation; (3) present in both *Eco*RI/*Hpa*II and *Eco*RI/*Msp*I (1,1), non-methylation; (4) absent in both *Eco*RI/*Hpa*II and *Eco*RI/*Msp*I (0,0), uninformative site.

Relative methylation/non-methylation levels were calculated as a ratio of the band number and the total bands for each pattern in the individual genotype and population. Significance difference between relative CG and CNG methylation levels and significance difference between relative total methylation and non-methylation levels were estimated by a Wilcoxon rank sum test [17] within each population. The relative CG, CNG methylation and non-methylation levels among natural populations were examined by a Kruskal–Wallis *H* test [43], respectively.

Shannon’s diversity index (*I*) was calculated to assess the epigenetic diversity (*H*) of the nine natural populations in SAS 9.2 system (Copyright 2008, SAS Institute Inc.) based on the frequency of different patterns in each polymorphism band among the 432 individuals. The formula was described as: *I* = – ∑ *P_i_* log_2_ (*P_i_*), where *P_i_* stands for the frequency of each 5′-CCGG methylation pattern. The index within each of the nine populations was defined as *H_pop_* and the index of the natural population was defined as *H_total_*. Significant differences in the Shannon’s index among populations were detected by the Kruskal–Wallis *H* test. Meanwhile, the significance test was adjusted by a sequential Bonferroni correction [46]. The coefficient of epigenetic differentiation was computed as *G_ST_* = (*H_total_* – *H_pop_*)/*H_total_* [47].

We transformed our data matrix into methylation-sensitive polymorphism (MSP matrix) and methylation pattern (CG-CNG matrix) profiles before multivariate analysis of epigenetic structure, which was performed with a ADE-4 software [48]. The transfer was conducted as below: MSP matrix, the methylation-sensitive polymorphism loci ((1) and (2)) were scored as “1″ and methylation-insensitive polymorphism patterns ((3) and (4)) were scored as “0″; CG-CNG matrix, hemi-methylation pattern (1) was defined as “1″ and full methylation pattern (2) was defined as “0″, while the methylation-insensitive polymorphism patterns were viewed as missing data [49]. A few synthetic variables were calculated to estimate the genome wide variability point of view based on principal component analysis (PCA) on inter-profile covariance matrix of MSP and CG-CNG, respectively.

For the natural populations, the between-populations variance was maximized by using a between-group eigen analysis (BPCA-PCA among groups based on PCA among individuals) [50], which divides the variance into within- and between-population components and is based on Euclidean distances. Therefore, a *β_ST_* value, analogous to *F*-statistics [51], which equals the ratio of the inertia between-population and the total inertia is generated. Also, Romesburg randomization test (9999 permutations) was used to detect the significance of differences between populations in the ADE-4 software [48].

The contribution of MSP and CG-CNG profiles to the natural population structures were evaluated by using a symmetrical co-inertia analysis, respectively. Statistical significance was assessed by 9999 Monte Carlo permutations in the ADE-4 software [48]. We also compared pairwise *β_ST_* (BPCA) values of the two profiles to assess their relationship [51].

## Supporting Information

Figure S1 **Forest form of the natural populations of** ***Populus tomentosa*** **located in Shandong Province (left), and xylem (shown by arrow) sampling location at 1.3 m height of the tree trunk (right).**
(TIF)
