# Supplementary figures and images for: Variation in Genomic Methylation in Natural Populations of Chinese White Poplar
Source: PLoS One. 2013 May 21;8(5):e63977. doi: 10.1371/journal.pone.0063977 (PMC3660595; doi:10.1371/journal.pone.0063977)

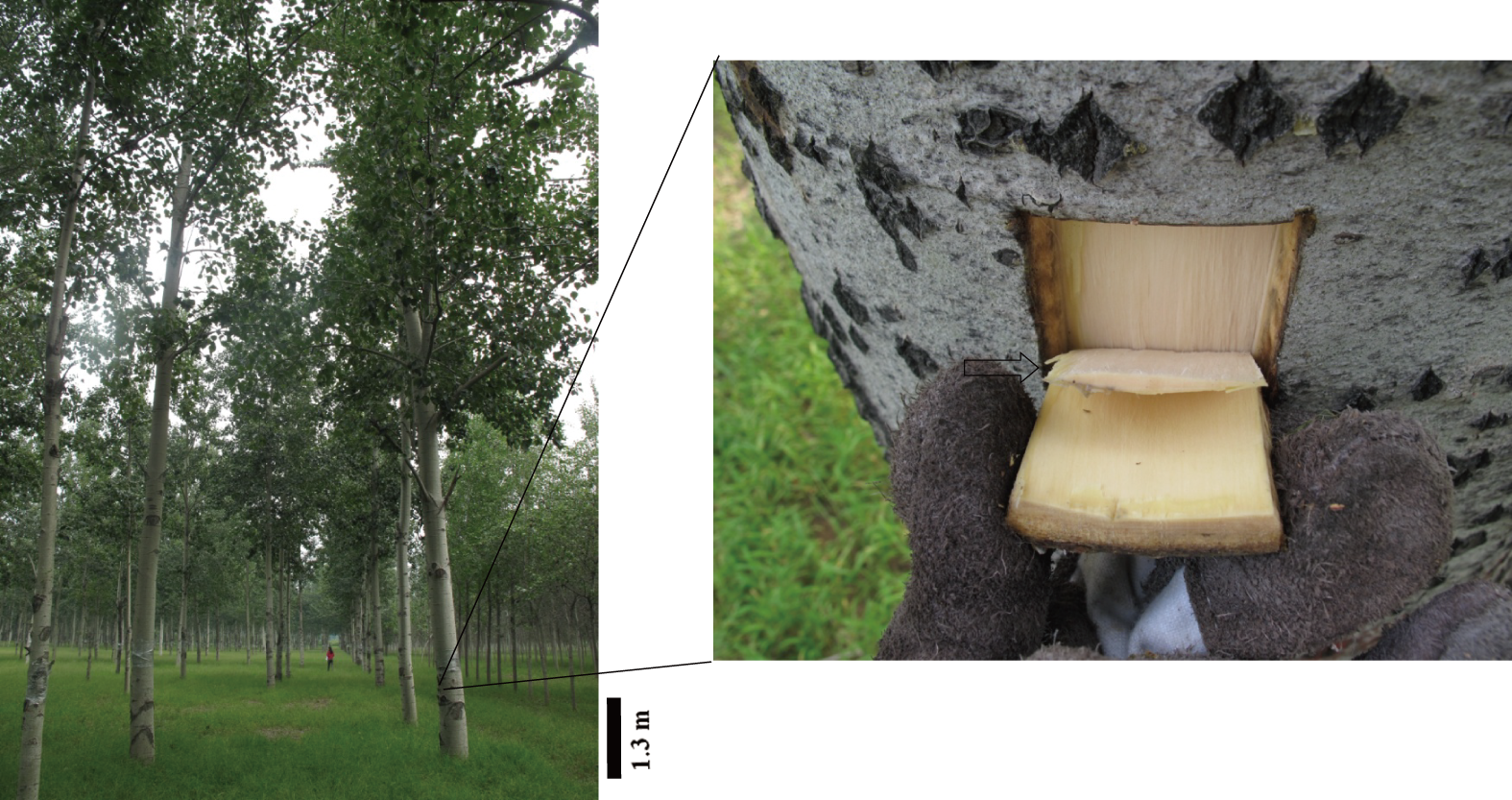

Supplement: Figure S1 — Forest form of the natural populations of Populus tomentosa located in Shandong Province (left), and xylem (shown by arrow) sampling location at 1.3 m height of the tree trunk (right). (TIF) [file pone.0063977.s001.tif]
